# Randomised controlled trial to evaluate the efficacy and usability of a computerised phone-based lifestyle coaching system for primary and secondary prevention of stroke

**DOI:** 10.1186/s12883-016-0540-4

**Published:** 2016-02-09

**Authors:** Lübomira Spassova, Debora Vittore, Dirk W. Droste, Norbert Rösch

**Affiliations:** 1 Luxembourg Institute of Science and Technology (LIST), 5, avenue des Hauts-Fourneaux, Esch/Alzette, 4362 Luxembourg; 2 Centre Hospitalier de Luxembourg – Service de Neurologie, 4, rue Barbé, Luxembourg, 1210 Luxembourg; 3 University of Applied Sciences Kaiserslautern – Campus Zweibrücken, Amerikastraße 1, Zweibrücken, 66482 Germany

**Keywords:** Stroke, Cerebrovascular, CVD, Prevention, Computer-supported, Phone-based, Clinical trial, Blood pressure, Nutrition, Usability

## Abstract

**Background:**

One of the most effective current approaches to preventing stroke events is the reduction of lifestyle risk factors, such as unhealthy diet, physical inactivity and smoking. In this study, we assessed the efficacy and usability of the phone-based Computer-aided Prevention System (CAPSYS) in supporting the reduction of lifestyle-related risk factors.

**Methods:**

A single-centre two-arm clinical trial was performed between January 2013 and February 2014, based on individual follow-up periods of six months with 94 patients at high risk of stroke, randomly assigned to an intervention group (IC: 48; advised to use the CAPSYS system) or a standard care group (SC: 46). Study parameters, such as blood pressure, blood values (HDL, LDL, HbA1c, glycaemia and triglycerides), weight, height, physical activity as well as nutrition and smoking habits were captured through questionnaires and medical records at baseline and post-intervention and analysed to detect significant changes. The usability of the intervention was assessed based on the standardised System Usability Scale (SUS) complemented by a more system-specific user satisfaction and feedback questionnaire.

**Results:**

The statistical evaluation of primary measures revealed significant decreases of systolic blood pressure (mean of the differences = –9 mmHg; *p* = 0.03; 95 % CI = [–17.29, –0.71]), LDL (pseudo-median of the differences = –7.9 mg/dl; *p* = 0.04; 95 % CI = [–18.5, –0.5]) and triglyceride values (pseudo-median of the differences = –12.5 mg/dl; *p* = 0.04; 95 % CI = [–26, –0.5]) in the intervention group, while no such changes could be observed in the control group. Furthermore, we detected a statistically significant increase in self-reported fruit and vegetable consumption (pseudo-median of the differences = 5.4 servings/week; *p* = 0.04; 95 % CI = [0.5, 10.5]) and a decrease in sweets consumption (pseudo-median of the differences = –2 servings/week; *p* = 0.04; 95 % CI = [–4, –0.00001]) in the intervention group. The usability assessment showed that the CAPSYS system was, in general, highly accepted by the users (average SUS score: 80.1).

**Conclusions:**

The study provided encouraging results indicating that a computerised phone-based lifestyle coaching system, such as CAPSYS, can support the usual treatment in reducing cerebro-cardiovascular risk factors and that such an approach is well applicable in practice.

**Trial registration:**

ClinicalTrials.gov Identifier: NCT02444715

## Background

Lifestyle risk factors, such as unhealthy diet, physical inactivity and smoking, have a strong influence on the personal risk of stroke. Reducing such risk factors is currently one of the most effective approaches to preventing stroke events. According to the World Health Organization (WHO), “most cardiovascular diseases can be prevented by addressing risk factors such as tobacco use, unhealthy diet and obesity, physical inactivity, high blood pressure, diabetes and raised lipids” [1–3]. However, many individuals have difficulties adhering to recommendations concerning a healthy lifestyle, and long-term compliance with such lifestyle changes is usually low. Early findings in behavioural research support the assumption that the mere task of self-monitoring increases habit awareness, induces reflection on habits, and thus can provoke a positive change of the monitored behaviour [4]. Furthermore, experience has shown that sustained contact is necessary in order to support people in establishing and maintaining lifestyle changes [5].

In this context, the recently developed Computer-aided Prevention System (CAPSYS) offers a cost-effective automated solution combining remote surveillance of individual cerebro-cardiovascular risk factors with tailored advice [6]. Registered patients can access CAPSYS through a phone interface, simply by dialling a landline number. In an automated dialog, patients are asked to provide information about a predefined number of risk factors (current nutrition, physical activity, smoking, weight and blood pressure) by typing in the corresponding answers using the phone keypad. Based on the patient’s answers, CAPSYS issues tailored feedback aiming at motivating a positive change towards or maintenance of healthy, risk-reducing habits. The system has been designed as a multi-language tool, in which the languages German and French have already been implemented and used within the trial. Clearly, a telephone as a user interface requires sufficient hearing abilities by the patients as well as the ability to understand and follow the instructions and advice issued by the virtual lifestyle coach. Consequently, this system is not appropriate for use by persons with severe hearing impairments or cognitive limitations. In cases of doubt, a patient’s cognitive abilities can be evaluated by means of the Folstein mini-mental state test [7]. On the other hand, there is no problem for patients with difficulties in articulation or speech impediments to use CAPSYS, due to the fact that patient interaction does not involve any speech input but is purely limited to pressing buttons on the telephone keypad.

In order to assess the efficacy of the CAPSYS approach concerning the reduction of stroke-related risk factors as well as the usability of the system, a dedicated study was designed and carried out, the results of which are presented in this article.

## Methods

In 2013 and 2014, a single-centre randomised controlled trial was carried out at the Department of Neurology of the Centre Hospitalier de Luxembourg. The study was designed as a six-month, parallel-group two-arm trial involving an intervention and a control group. Within this six-month study with a relatively limited number of participants, it was not possible to focus on the registration of severe events, such as stroke or death. Instead, the study concentrates on changes in different surrogate parameters for stroke (systolic blood pressure, BMI, HDL, LDL, HbA1c, glycaemia and triglycerides). A draft of the study design has already been presented in [8].

### Participants

Patients aged 20 and over were eligible for the study if they had already suffered from a stroke or were at high risk of stroke (at least two risk factors according to the criteria described in [2, 9, 10]). The eligibility criteria are summarised in Table 1. Patients’ stroke risk and thus their suitability to participate in the CAPSYS study were evaluated by the treating neurologist based on the patients’ individual risk factor profiles.

**Table 1:** Eligibility criteria for the CAPSYS study

Inclusion criteria	Exclusion criteria
Age: 20 and over; At high risk of stroke:	Inability to fill out or to understand the informed consent;
∙ Already suffered a stroke or Transient Ischemic Attack (TIA) or	No signed informed consent;
∙ At least two risk factors for stroke:	
– High blood pressure (systolic blood pressure ≥ 140 *mmHg*)	Dementia
– Overweight (BMI ≥ 25 *kg*/*m*^2^)	
– Low physical activity (less than 30 min. of moderate-intensity physical exercise per week)	
– Smoking	
– Unhealthy diet	

### Recruitment and randomisation

Potential study participants were approached in the course of their regular consultations with their neurologist, where they were screened for eligibility. Prior to their enrolment in the study, all participants signed a written informed consent; 94 participants were randomised by lot to one of two groups: Interventional Care (IC: 48, 34 [71 %] male) or Standard Care (SC: 46, 29 [63 %] male). Participants were aged between 32 and 86 (SC: mean = 59.6; SD = 12.1; median = 59) (IC: mean = 60.7; SD = 11.3; median = 61). The major characteristics of the study cohort are summarised in Table 2. Due to the specific design of the intervention, the study had to be carried out in an only partially blinded manner. It is inherent to the CAPSYS approach that the treating neurologist performs regular reviews of the data collected in the intervention group. The need to have access to patient histories and the possibility to contact patients at any time in case of emerging situations has limited the use of blinding methods within the trial. However, in order to ensure the validity of the study, the CAPSYS study team was separated into three different groups: a) principal investigator and treating neurologist responsible for the initial eligibility check of potential study participants, b) hospital research nurse responsible for patient recruitment and randomisation, and c) healthcare researchers who performed the statistical evaluation of the collected data by using pseudonymised data sets. During the recruitment meeting, patients were informed about the purpose of the study and about the study approach. The CAPSYS study design was approved by the Luxembourg National Research Ethics Committee (CNER) (N° 201205/08) and the National Commission for Data Protection (CNPD) (T007990).

**Table 2:** Descriptive statistics of the study cohort

	SC (*n* = 46)	IC (*n* = 48)
Mean age [*years*] (±SD)	59.6 (±12.1)	60.7 (±11.3)
Men	29 (63 %)	34 (71 %)
Mean BMI [ *kg*/*m*^2^] (±SD)	27 (±4.3)	28 (±4.3)
Smokers	2 (4 %)	6 (13 %)
Hypertension	36 (78 %)	43 (90 %)
Hyper-/Dyslipidemia	39 (85 %)	44 (92 %)
Diabetes mellitus	5 (11 %)	8 (17 %)
Previous stroke/TIA	37 (80 %)	31 (65 %)

### Intervention

In addition to the usual care, participants in the IC group were advised to regularly call the phone-based prevention system CAPSYS (preferably twice a week). In the course of the recruitment process, they were carefully instructed in the proper use of the system, and they were provided a corresponding user manual in form of a leaflet.

The participants could access the CAPSYS system by calling a specific land line number with their stationary or mobile phones. After choosing their preferred language (French or German) and authenticating themselves with their individual patient number and PIN code, the participants were posed the following questions:

- How many servings of fruits and vegetables have you consumed yesterday?
- How many servings of whole-grain food have you consumed yesterday?
- How many servings of sweets have you consumed yesterday?
- Please enter your current weight.
- How many cigarettes have you smoked yesterday? [removed for non-smokers]
- How many minutes have you been physically active yesterday? [moderate or high intensity]
- Please enter your current systolic blood pressure.

The participants could answer to these questions by typing in the numerical values (number of servings, weight, minutes etc.) using the phone key pad. During the recruitment meeting at the beginning of the study, participants were instructed on how to estimate the size of one portion for the relevant food groups, and they were advised to measure their current blood pressure and weight, and to write down all necessary values prior to calling the CAPSYS system.

Depending on the data provided by the patient, he or she received immediate customized feedback after each answer concerning his or her progress and advice on how to proceed reducing the corresponding risk factor or motivation on maintaining some observed healthy behaviour respectively. The phone call was closed with a concluding statement summing up the feedback issued before by the system. The questions and feedback were automatically generated by CAPSYS and were issued to the patients in spoken natural language by means of a text-to-speech software (TTS). The system was implemented to run fully automatically and could be accessed at any time of day.

In case of systolic blood pressure higher than 140 mmHg, the patient was advised to rest for a while and then repeat the measurement. The system also advised the patients to contact a doctor in case of constantly high blood pressure. Patients with values above 140 mmHg were also particularly marked in the web interface when the treating physician accessed the system. Apart from that, all participants have been informed that there were no direct alerts in cases of high blood pressure provided within the study.

The treating neurologist could access the data provided by his patients through a web interface, offering both a numerical as well as an adaptable graphical representation of the patient data for a certain period of time. The use of CAPSYS within the study has not replaced or reduced the existing information exchange between the neurologist and the corresponding referring physicians, which was proceeded according to the practices of the Luxembourgish healthcare system. Technically, CAPSYS offers the possibility for further physicians, e.g. the treating general practitioner, to access and monitor the data gathered from their patients. However, in the scope of this study, this option was deliberately discarded in order to avoid potential bias caused by the general practitioner accessing and interpreting the CAPSYS data.

In contrast to the IC group, SC participants received only the usual care including blood analyses, blood pressure controls and individual advice on healthy lifestyle during the outpatient treatment given by the neurologist, by the general practitioner and by other physicians. Information material concerning a healthy lifestyle was provided to both groups.

### Data collection

Demographic and medical information of each participant was recorded by the study personnel in a predefined form, including body weight and height, medical history, current medication, smoking habits, blood pressure and heart rate as well as recent blood test results (HDL, LDL, HbA1c, glycaemia and triglycerides). Furthermore, participants were asked to fill in three questionnaires exploring their nutritional habits, physical activity and quality of life. The latter was measured using the standardised EQ-5D-5L instrument provided by the EuroQol Group [11].

Six months after enrolment, participants were asked to fill in the same questionnaires again, and current medical data (weight, blood pressure, heart rate, blood test results, smoking habits and medication) were again recorded by the study personnel. In addition, IC participants were asked to share their experiences with using CAPSYS and to provide their opinions about the system in a predefined questionnaire, which encompasses the well-established System Usability Scale (SUS) [12] and some further more specific questions concerning the usage of CAPSYS and its particular features. There was also space left for open-ended comments and suggestions at the end of the questionnaire.

### Dependent measures

The impact of the intervention was assessed based on the changes of cerebro-cardiovascular risk parameters during the six-month trial period, whereby changes in the IC group were compared to those in the control group (SC). Changes in systolic blood pressure, HDL, LDL, HbA1c, glycaemia, triglycerides and BMI were considered as primary dependent measures (see Table 3). Changes in self-reported weekly consumption of fruits and vegetables, whole grain food and sweets as well as changes in self-reported weekly duration of physical activity and quality of life were analysed as secondary measures. Due to the low number of smokers among the study participants (SC: 2, IC: 6), smoking habits were not evaluated.

**Table 3:** Overview of baseline values and evaluation of primary dependent measures in SC and IC groups

	SC		IC	
	Baseline	Evaluation	Baseline	Evaluation
*Parametric data*	*Mean (±SD)*	*T-test*	*Mean (±SD)*	*T-test*
Systolic BP [*mmHg*]	139.6 (±21)	mean of diff. =0.19	145 (±19.4)	mean of diff. =−9
		*p* =0.96		***p*** = 0.03*
		95 % CI = [–7.5, 7.88]		95 % CI = [–17.29, –0.71]
				Cohen’s d =0.42
BMI [ *kg*/*m*^2^]	27 (±4.3)	mean of diff. =0.03	28 (±4.3)	mean of diff. =−0.18
		*p* =0.9		*p* =0.43
		95 % CI = [–0.49, 0.55]		95 % CI = [–0.65, 0.28]
*Non-parametric data*	*Median [IQR]*	*Wilcoxon*	*Median [IQR]*	*Wilcoxon*
HDL [ *mg*/*dl*]	57 [45, 51]	pseudo-median of diff =1.5	53 [47, 74]	pseudo-median of diff =−1.5
		*p* =0.53		*p* =0.44
		95 % CI = [–2.5, 4.5]		95 % CI = [–4.5, 2]
LDL [ *mg*/*dl*]	96 [71, 114]	pseudo-median of diff =−1	95 [76, 117]	pseudo-median of diff =−7.9
		*p* =0.76		***p*** = 0.04*
		95 % CI = [–10.5, 6]		95 % CI = [–18.5, –0.5]
				r =0.24
HbA1c [ *%*]	5.8 [5.6, 5.9]	pseudo-median of diff =0	5.9 [5.5, 6.25]	pseudo-median of diff =0.1
		*p* =0.92		*p* =0.37
		95 % CI = [–0.25, 0.2]		95 % CI = [–0.1, 0.25]
Glycaemia [ *mg*/*dl*]	98 [89, 106.75]	pseudo-median of diff =0.5	102 [92.5, 114.5]	pseudo-median of diff =2.5
		*p* =0.78		*p* =0.19
		95 % CI = [–5.5, 5]		95 % CI = [–2, 6]
Triglycerides [ *mg*/*dl*]	95 [73, 134.25]	pseudo-median of diff =−6	105 [78, 132.5]	pseudo-median of diff =−12.5
		*p* =0.39		***p*** = 0.04*
		95 % CI = [–21, 10]		95 % CI = [–26, –0.5]
				r =0.25

(statistically significant values are marked in bold)

Statistical analyses were carried out on an intention-to-treat basis using RStudio (Version 0.98.978) with a significance level of 0.05. Parametric data (paired and unpaired samples) were evaluated with the Student’s t-test approach, and the corresponding effect size was calculated with Cohen’s d. Non-parametric data were evaluated using the Wilcoxon signed-rank test (for paired samples) or the Mann-Whitney U test (for unpaired samples) respectively, and effect size was calculated as $r = \frac {Z}{\sqrt {N}}$. For non-parametric paired samples, the pseudo-median is computed as the Hodges-Lehmann estimate.

## Results

### Efficacy of the intervention

Final questionnaires on nutritional habits, physical activity and quality of life could be collected from 68 participants (SC: 36; IC: 32) at the end of the study. This results in an overall dropout rate of 28 % (SC: 22 %; IC: 33 %). Further participant data, such as blood test values, could be retrieved from medical records. Out of the 48 recruited IC participants, 11 (23 %) did not use CAPSYS at all. The remaining 37 IC subjects (77 %) called the system up to 55 times during the study period (mean = 23.32; SD = 14.1; median = 24).

**Fig. 1:**
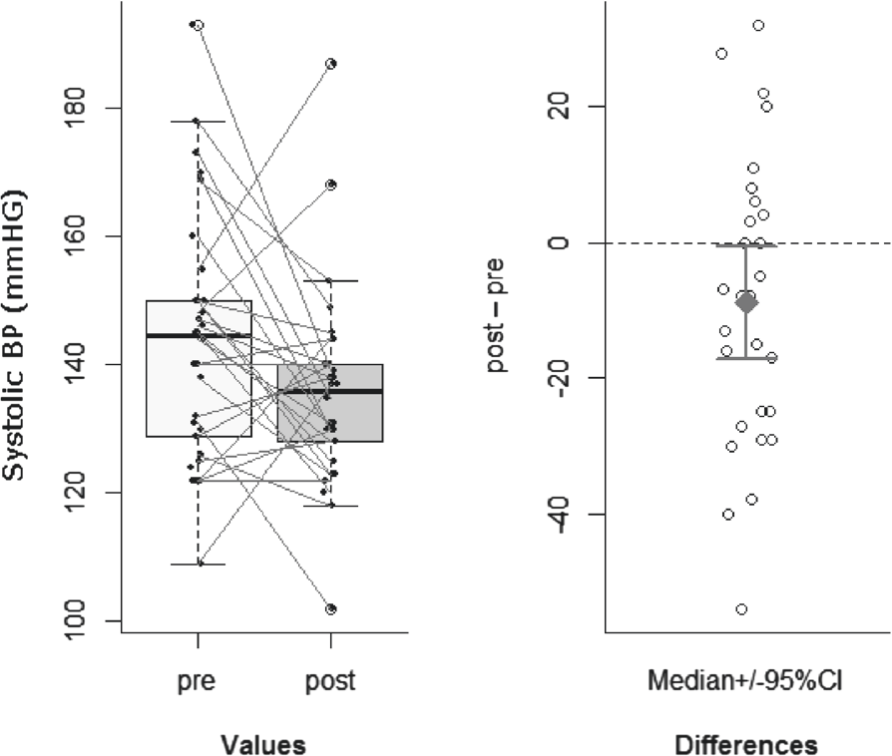
Systolic blood pressure. Significant decrease of systolic blood pressure in IC group
- Decrease of *low-density lipoprotein (LDL) levels* in IC group (Wilcoxon signed-rank test: pseudo-median of the differences = –7.9 mg/dl; *p* = 0.04; 95 % CI = [–18.5, –0.5]; r = 0.24: medium effect size), with no corresponding significant difference in SC group (*p* = 0.76) (see Fig. 2);

Within-subjects analyses of primary dependent measures showed the following statistically significant changes between baseline and post-intervention:

- Decrease of *systolic blood pressure* in IC group (two-sided paired-samples t-test: mean of the differences = –9 mmHg; *p* = 0.03; 95 % CI = [–17.29, –0.71]; Cohen’s d = 0.42: large effect size) (see Fig. 1), with no corresponding significant difference in SC group (*p* = 0.96);

**Fig. 2:**
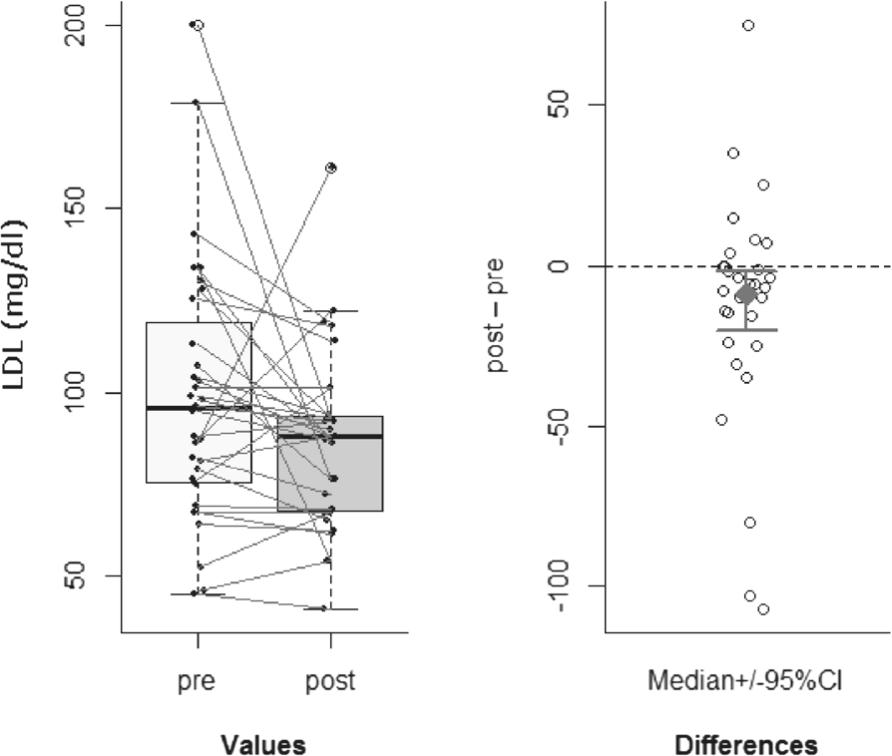
LDL. Significant decrease of LDL levels in IC group
- Decrease of *triglyceride levels* in IC group (Wilcoxon signed-rank test: pseudo-median of the differences = –12.5 mg/dl; *p* = 0.04; 95 % CI = [–26, –0.5]; r = 0.25: medium effect size), with no corresponding significant difference in SC group (*p* = 0.39) (see Fig. 3);

**Fig. 3:**
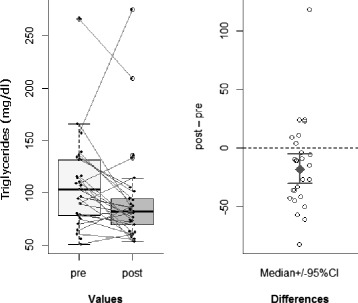
Triglycerides. Significant decrease of triglyceride levels in IC group
- No statistically significant changes could be found for any of the other primary measures (*BMI*, *HDL*, *HbA1c* and *glycaemia*).

In this context, it needs to be stated that, at baseline, there were no statistical differences between both groups concerning the proportions of patients taking antihypertensives (SC: 36 [78 %]; IC: 43 [90 %]; *p* = 0.22), antidiabetic drugs (SC: 5 [11 %]; IC: 8 [17 %]; *p* = 0.61) or cholesterol-lowering medication (SC: 39 [85 %]; IC: 44 [92 %]; *p* = 0.47).

Concerning the secondary dependent measures, we could observe the following statistically significant results:

- Increase of *self-reported fruit and vegetable consumption* in IC group (Wilcoxon signed-rank test: pseudo-median of the differences = 5.4 servings/week; *p* = 0.04; 95 % CI = [0.5, 10.5]; r = 0.26: medium effect size), with no corresponding significant difference in SC group (*p* = 0.67);
- Decrease of *self-reported sweets consumption* in IC group (Wilcoxon signed-rank test: pseudo-median of the differences = –2 servings/week; *p* = 0.04; 95 % CI = [–4, –0.00001]; r = 0.26: medium effect size), with no corresponding significant difference in SC group (*p* = 0.06);
- Difference between SC and IC groups concerning the change of *self-reported whole-grain food consumption* (Mann-Whitney U test: difference in location = 3.26 servings/week; *p* = 0.04; 95 % CI = [0.00006, 7]; r = 0.24: medium effect size);
- No statistically significant changes for *self-reported physical activity* and *quality of life*.

### Usability and user acceptance of the intervention

Assessments of the intervention based on the System Usability Scale (SUS) were provided by 28 participants of the IC group, 2 of which were declared invalid, as these participants have demonstrably never used CAPSYS during the trial period, and consequently, they were considered unable to assess the intervention. The average SUS score for CAPSYS was 80.1 (SD = 16.22; median = 78.75; min = 37.5; max = 100; 95 % CI = [73.54, 86.65]). According to Sauro and Lewis, this average score corresponds to an “A–” school grade and a percentile of 85–89, meaning that CAPSYS scored better than 85 to 89 % of all systems considered in the Sauro and Lewis study (see [13], chapter 8, page 204, table 8.6). Using the simplified regression equation proposed by Lewis [14], the Likelihood to Recommend for each CAPSYS user was derived from the corresponding SUS scores as LTR = SUS/10. With these values, we could identify 10 promoters (38 %; LTR ≥ 9) and 4 detractors (23 %; LTR ≤ 6) among the 26 participants who provided valid SUS scores, which results in a positive Net Promoter Score (NPS) of 23 [15], meaning that there are 23 % more users who would recommend CAPSYS (promoters) than users who would argue against using the system (detractors).

Beside the standardised SUS questionnaire, IC participants were also asked to asses the voice of the text-to-speech module applied in CAPSYS and the automatically generated utterances issued by the system. The results concerning these questions are presented in Table 4.

**Table 4:** Evaluation of CAPSYS’s voice and utterances

	Scale	Results		
Voice	1: pleasant – 5: unpleasant	mean =1.77;	median =1.5;	SD =0.99
	1: difficult to understand – 5: easy to understand	mean =4.28;	median =5;	SD =1.14
	1: natural – 5: unnatural	mean =2.15;	median =2;	SD =1.12
	1: too fast – 5: too slow	mean =3.2;	median =3;	SD =0.5
Utterances	1: diversified – 5: unvaried	mean =3.28;	median =4;	SD =1.24
	1: inappropriate – 5: appropriate	mean =3.46;	median =3;	SD =1.07
	1: too short – 5: too long	mean =3.2;	median =3;	SD =0.5

Concerning the frequency of use, the majority of the 26 IC participants who answered to the usability questionnaire indicated that they would prefer using CAPSYS once (11; 42 %) or twice (9; 35 %) per week. None of the respondents was willing to use the system more than twice a week, 2 (8 %) preferred to use it less than once a week and 4 (15 %) expressed that they would not use it at all. The approach of connecting to CAPSYS by calling the system whenever they wish was favoured by most of the respondents (18; 69 %) over the potential alternatives of being called by the system (3; 12 %) or receiving reminders to call the system (2; 8 %). Although CAPSYS does not offer speech recognition, we asked users if they prefer to have such an input option instead or in combination with the implemented keypad input. In this context, using the phone keypad was identified as the most preferred way of communication with such a system (20; 77 %) as compared to speech input (1; 4 %) or a combination of both (4; 15 %). The majority of the respondents were interested in having insight into the data history gathered by CAPSYS, either online (9; 35 %) or in a printed version (11; 42 %). Concerning a potential alternative interface for CAPSYS beside the phone interface, 10 (38 %) participants were interested in a corresponding website and 4 (15 %) fancied the idea of having a CAPSYS smartphone app. Regarding the question if the use of CAPSYS has motivated them to a healthier lifestyle, 5 (19 %) of the respondents stated that through the intervention they started eating healthier, 3 (12 %) reported to have become physically more active and 12 (46 %) claimed to have achieved both healthier eating and increased physical activity. This means that, in total, 20 (77 %; 95 % CI = [59 %, 95 %]) of the respondents expressed an improvement of their health-related lifestyle (improved nutrition or increased physical activity or both) through the use of CAPSYS.

## Discussion

The positive effects that could be observed in the IC group concerning systolic blood pressure, LDL and triglyceride levels as well as the self-reported improvements of nutritional habits can be regarded as an indicator that a computerised phone-based intervention, such as CAPSYS, can support the reduction of cerebro-cardiovascular risk when offered in addition to usual care. An average decrease in systolic blood pressure of 9 mmHg among CI participants can be regarded as clinically significant [16]. The same also holds for the observed average decreases of 7.9 mg/dl for LDL [17] and 12.5 mg/dl for triglycerides [18]. Though, the relatively wide 95 % confidence intervals retrieved in the statistical analysis indicate that the cohort size was probably too small to be able to demonstrate strong clinical effects. The evaluation of BMI, HDL, HbA1c and glycaemia values showed no significant changes in the two groups.

Our results are to some extent supported by the recently published Australian CLIP study, which found that telephone coaching performed by human health care professionals can result in a significant reduction of LDL and total cholesterol values in the intervention group [19]. A systematic review by Goode et al. also underlines the efficacy of telephone-based lifestyle coaching. In 20 out of 26 studies, telephone support by health professionals have led to significant improvements concerning physical exercises and healthy nutrition [20]. However, personal telephone coaching by health experts requires intensive use of human resources and is associated with enormous costs, which limits its widespread distribution to larger patient groups.

CAPSYS is a system that supports prevention initiatives by supporting individuals in daily life settings. It can be assumed that a large percentage of the total target group is not willing to perform any lifestyle changes at all, no matter which kind of support they will receive. So, it has to be taken into account that our study participants are a selected subgroup of the target population with a potentially higher motivation to change risk factors and lifestyle. However, this selection bias in general concerns all prevention initiatives, and the randomisation process should ensure that the general motivation is balanced across both study arms.

Compared to therapy studies, participants in prevention studies mostly do not see any direct measurable advantages in their actual health status. Missing short-term improvements are therefore often a reason for higher dropout rates. However, compared to other studies in secondary prevention, the moderate and balanced dropout rates of both CAPSYS arms should not have significantly affected the study results. On the other hand, self-reported consumptions of fruits, vegetables or sweets have a high potential for reporting bias. Therefore, in our study, this kind of information is only considered as secondary depended measures.

The improvements of nutritional habits in the IC group (5 servings of fruits and vegetables more per week; 2 servings of sweets less per week) are significant, but have to be treated with caution as they are based on self-reported values and thus less reliable. As the dropout rates were not significantly different for both groups (SC: 22 %; IC: 33 %; *p* = 0.31), it can be supposed that the withdrawal of IC patients from the clinical trial was not substantially influenced by the intervention itself, but it was due to common reasons, like lack of time, forgetting appointments or just lack of motivation. Furthermore, it remains open how the observed positive effects will evolve after the study period and if they actually are of a long-term nature.

The usability evaluation revealed that the CAPSYS approach was well accepted by the study participants. In particular, the large proportion of respondents (77 %) expressing a positive change of health-related behaviour due to the use of CAPSYS is remarkable. One might argue that this result is strongly biased due to the fact that users who have a positive experience with a system are more likely to finish a study and provide corresponding feedback than users with negative experiences. However, even if we consider all dropout IC participants as non-beneficiaries, the success rate concerning a positive health behaviour change would still be 42 % (95 % CI = [27 %, 57 %]). Unfortunately, our cohort was not large enough to perform subgroup analyses in order to evaluate the characteristics of beneficiaries and non-beneficiaries. A follow-up study focussing on such aspects might help to identify appropriate target groups for which a system like CAPSYS might be most beneficial and most accepted.

During informal interviews at the end of the study, many of the IC participants explained that they often could not find the time or simply forgot to call CAPSYS. Taking this into account, the implementation of a call reminder functionality seems beneficial, even though in the feedback questionnaire, the majority of the respondents did not favour the concepts of being called or reminded by the system. Another important issue concerning risk factor reduction in the stroke prevention context is the topic of medication adherence. In order to support users in regularly taking their antihypertensive or cholesterol-lowering medication, CAPSYS could be extended to incorporate prompts in its automatically generated feedback messages asking the users if they have taken their prescribed medication according to their individual medication plan.

Concerning the technical implementation of CAPSYS, it could be observed that, on average, the artificial voice applied in CAPSYS was perceived as rather pleasant, easy to understand and natural, and the speech rate was perceived as appropriate. The length of the utterances was, on average, perceived as adequate, but the feedback text might also be shortened to some extent. The results also show that there is some space for improvement concerning the diversity and adequacy of the automatically generated feedback.

The offered keypad interface for data input was highly accepted by the users, and the need for an alternative input method, such as speech, was detected to be low. Unsurprisingly, only few users were currently interested in a mobile app version of CAPSYS. However, with increasing spread of smartphones, a mobile solution will certainly gain in importance in the future. What is already important for most users is to have access to their own self-monitored data, even if due to technical reasons they might only be able to access them in a printed form.

## Conclusion

In summary, the present study provided encouraging results indicating that a computerised phone-based lifestyle coaching system, such as CAPSYS, can support the usual treatment in reducing cerebro-cardiovascular risk factors and that such an approach is in general well accepted by the affected patients. In a next step, CAPSYS could be extended to a disease management tool, involving health professionals from different disciplines, such as neurologists, cardiologists, dieticians, physiotherapists etc., in the treatment of patients. One aspect that could be explored in future research is if the motivational effect of CAPSYS could be strengthened through an increased involvement of the treating physician and through incorporation of social interaction.
